# Gene expression profiling and in vitro functional studies reveal *RAD54L* as a potential therapeutic target in multiple myeloma

**DOI:** 10.1007/s13258-022-01272-7

**Published:** 2022-06-11

**Authors:** Ivyna Pau Ni Bong, Ching Ching Ng, Norodiyah Othman, Ezalia Esa

**Affiliations:** 1 Haematology Unit, Cancer Research Centre, Institute for Medical Research, National Institutes of Health, Ministry of Health Malaysia, 40170 Shah Alam, Selangor Malaysia; 2 Faculty of Science, Institute of Biological Sciences, University of Malaya, Kuala Lumpur, Malaysia

**Keywords:** Multiple myeloma, Differentially expressed genes, *RAD54L*, Cell proliferation, Apoptosis, Cell cycle

## Abstract

**Background:**

Current advances in the molecular biology of multiple myeloma (MM) are not sufficient to fully delineate the genesis and development of this disease.

**Objective:**

This study aimed to identify molecular targets underlying MM pathogenesis.

**Methods:**

mRNA expression profiling for 29 samples (19 MM samples, 7 MM cell lines and 3 controls) were obtained using microarray. We evaluated the in vitro effects of *RAD54L* gene silencing on the proliferation, apoptosis and cell cycle distribution in KMS-28BM human MM cells using siRNA approach. Cell proliferation was determined by MTS assay while apoptosis and cell cycle distribution were analysed with flow cytometry. Gene and protein expression was evaluated using RT-qPCR and ELISA, respectively.

**Results:**

Microarray results revealed a total of 5124 differentially expressed genes (DEGs), in which 2696 and 2428 genes were up-regulated and down-regulated in MM compared to the normal controls, respectively (fold change ≥ 2.0; *P* < 0.05). Up-regulated genes (*RAD54L*, *DIAPH3*, *SHCBP1*, *SKA3* and *ANLN*) and down-regulated genes (*HKDC1*, *RASGRF2*, *CYSLTR2*) have never been reported in association with MM. Up-regulation of *RAD54L* was further verified by RT-qPCR (*P* < 0.001). In vitro functional studies revealed that *RAD54L* gene silencing significantly induced growth inhibition, apoptosis (small changes) and cell cycle arrest in G0/G1 phase in KMS-28BM (*P* < 0.05). Silencing of *RAD54L* also decreased its protein level (*P* < 0.05).

**Conclusions:**

This study has identified possible molecular targets underlying the pathogenesis of MM. For the first time, we reveal *RAD54L* as a potential therapeutic target in MM, possibly functioning in the cell cycle and checkpoint control.

**Supplementary Information:**

The online version contains supplementary material available at 10.1007/s13258-022-01272-7.

## Introduction

Multiple myeloma (MM) is a cancer of plasma cells. It is a highly heterogeneous and genetically complex form of blood cancer (Corre et al. 2015). MM is the second leading cause of haematological malignancy in the world (de Mel et al. 2014). Development and progression of MM required multiple primary and secondary oncogenic events. Hyperdiploidy and translocations concerning the IgH locus are frequent primary genetic events in the initiation of MM disease. IgH translocations frequently lead to the activation of proto-oncogenes for instance *FGFR3*/*MMSET*/t(4;14), *CCND1*/t(11;14), *c-MAF*/*CCND2*/t(14;16), *MAF-B*/t(14;20) and *CCND3*/t(6;14) (Colombo et al. 2015; Pinto et al. 2020). Acquired mutations (*K-RAS*, *N-RAS*, *BRAF*, *DIS3*, *FAM46C* and *TP53* genes), copy number aberrations (del(17p)/TP53, del(13) and 1q gain), NF-κB pathway mutation and *MYC* aberrations are recurrent secondary oncogenic events in MM, which are associated with disease progression (Colombo et al. 2015; Roy et al. 2018; Pinto et al. 2020).

Current advances in research have dramatically expanded our understanding of the genomic landscape, tumour heterogeneity and clonal evolution in MM (Furukawa and Kikuchi 2020). However, MM remains incurable with a poor prognosis due to a lack of suitable tumour markers for early diagnosis and treatment. Therefore, identifying new tumour biomarkers and therapeutic targets are essential steps to improve the prognostic and outcomes of patients. The aims of this study are to identify molecular targets underlying the pathogenesis of MM by mRNA expression profiling and functional target validation by using siRNA and cell-based assays.

## Materials and methods

### Patient samples

Bone marrow or whole blood were taken from MM patients (N = 19: MM1-MM19) and healthy donors (N = 3: NB1, NB2, and NB3) and stored at − 80 °C. The average age of the patient was 57 years while the median age was 61 years. The age of the patients was in the range of 28–74 years. Seventeen patients were newly diagnosed MM (NDMM), 2 were from relapsed MM cases. Patients were with > 10% plasma cell infiltration at the time of sample recruitment. Cytogenetic analysis indicated 13 patients with normal karyotype, 1 patient with hypodiploidy and 5 patients with unknown karyotype. Patients’ clinical parameters are shown in Table 1.

**Table 1:** Clinical parameters for 19 multiple myeloma patients

Patient ID	Age at diagnosis	Race	Gender	Cytogenetic analysis	NDMM/relapsed MM
MM1	68	Malay	F	No chromosomal abnormality observed	NDMM
MM2	30	Others	M	No chromosomal abnormality observed	NDMM
MM3	64	Malay	F	No chromosomal abnormality observed	NDMM
MM4	74	Chinese	M	No chromosomal abnormality observed	NDMM
MM5	59	Malay	M	No chromosomal abnormality observed	NDMM
MM6	48	Others	M	No chromosomal abnormality observed	NDMM
MM7	28	Malay	M	No chromosomal abnormality observed	NDMM
MM8	48	Malay	F	Unknown	NDMM
MM9	62	Malay	F	Unknown	NDMM
MM10	64	Malay	F	No chromosomal abnormality observed	Relapsed MM
MM11	61	Chinese	M	Unknown	Relapsed MM
MM12	70	Malay	F	No chromosomal abnormality observed	NDMM
MM13	72	Others	M	No chromosomal abnormality observed	NDMM
MM14	51	Others	F	No chromosomal abnormality observed	NDMM
MM15	65	Malay	F	Hypodiploidy with multiple abnormalities	NDMM
MM16	61	Chinese	M	No chromosomal abnormality observed	NDMM
MM17	51	Others	M	Unknown	NDMM
MM18	48	Malay	M	Unknown	NDMM
MM19	62	Malay	F	No chromosomal abnormality observed	NDMM

*MM* multiple myeloma, *NDMM* newly diagnosed multiple myeloma

### Cell lines

The U-266, RPMI-8226, IM-9 and MM.1S cells were obtained from American Type Culture Collection (ATCC, USA). The KMS-28BM, KMS-12BM and KMS-20 were purchased from the Japanese Collection of Research Bioresources (JCRB) cell bank. Multiple myeloma cells were cultured and maintained in RPMI-1640 medium (PAN-Biotech, Germany) with 10% fetal bovine serum (Sigma-Aldrich, Germany) in an incubator at 37 ℃ with 5% CO_2_. Cells were sub-cultured when achieved 70–75% confluence. Cells at the logarithmic phase were used for transfection.

### Isolation of total RNAs

Total RNAs were isolated from the bone marrow/peripheral blood/cells according to manufacturer’s recommendation (Qiagen RNeasy mini kit). DNA digestion was performed to ensure that the RNA was free of DNA contamination (Qiagen DNase I, Hilden, Germany). The integrity of the total RNAs used for gene expression microarray were checked with Bioanalyser (RNA Nano Chip, Agilent 2100 Bioanalyser). All samples included in the gene expression microarray had an RNA integrity number (RIN) of at least 8.0. The purity of the isolated Total RNAs was also checked with NanoDrop ND-1000 UV–VIS spectrophotometer to ensure that the purity was within the range of 1.80–2.10.

### Gene expression microarray assay

Sample processing, labelling, and hybridisation were performed following the standard protocol recommended by Agilent’s manufacturer. Approximately 60,000 probes were contained on each array. Briefly, 100 ng of total RNAs were labelled using one colour Agilent’s Low Input Quick Amp Labelling kit and purified with spin column (Qiagen RNeasy mini kit, Hilden, Germany). Hybridisation was performed using 600 ng of labelled cRNAs onto SurePrint G3 Human GE 8 × 60 K V2 Microarray Kit (Agilent Technologies, USA). The microarray slide was then put into an incubator with rotation at 65 °C for 17 h. Microarray images were scanned and data from raw microarray image files were extracted with Agilent Feature Extraction Software Version 10.7.3.1 to prepare for analysis. Only samples that passed the raw data quality control metrics as described by Agilent’s recommended procedure were proceeded to data analysis. Pre-processing of the data files in this study was performed by using GeneSpring software version 14.9. All the raw data were thresholded to 1 and normalised to the 75th percentile. This was followed by a baseline transformation set to the median of all samples.

Significantly differentially expressed probes in MM vs. normal controls were identified by unpaired unequal variance *t-*test (Welch) (*P* < 0.05). The Benjamini Hochberg false discovery rate (FDR) multiple testing corrections was used to identify differentially expressed probes. The resulting list was further refined by analysing it to a second filter, which specified a 2.0-fold change between MM vs. controls. Only probes that passed a p-value cut-off of 0.05 and fold change ≥ 2.0 were considered significant. Unsupervised hierarchical clustering analysis was carried out for the up-regulated and down-regulated probes, respectively, with a p-value cut-off of 0.05 and fold change ≥ 2.0. Unsupervised hierarchical clustering was generated using Euclidean distance metric and average linkage statistical methods.

## In vitro functional study of *RAD54L* in KMS-28BM human MM cell line

### siRNA transfection

Three target-specific siRNA oligo duplexes (Cohesion Biosciences, Catalog No.: CRH5528) of the human *RAD54L* gene were pooled together to knockdown the target gene in KMS-28BM MM cells. Briefly, 2 × 10^6^ cells were resuspended in 100 μl of 4D-Nucleofector™ solution (Lonza, USA) and mixed with 500 nM of *RAD54L* siRNA or negative control siRNA. The mixture was transferred to a cuvette. Transfection was then carried out using program DY-100 in a 4D-Nucleofector™ system (Lonza, USA). Then, cells in the cuvette were gently transferred to warm medium in 24 well plates. Two or three independent experiments were carried out for each transfection.

### RT-qPCR analysis

Total RNAs were converted to first strand cDNA following manufacturer’s protocol (High Capacity RNA-to-cDNA kit, Applied Biosystems, USA). The RT-qPCR was performed using the TaqMan gene expression assay (Applied Biosystems, USA) in the StepOnePlus™ Real-time PCR System (Applied Biosystems, USA). Pre-designed TaqMan gene expression assays for *RAD54L* (Hs00936473_m1, ThermoFisher Scientific, USA) and internal control *GAPDH* (Hs02758991-g1, ThermoFisher Scientific, USA) were used. Thermal cycling conditions consisted of the following: initial denaturation at 95 °C for 20 s, followed by 40 cycles of 95 °C for 1 s and 60 °C for 20 s. The relative expression of genes was calculated and quantified based on 2^−ΔΔCt^ method.

### MTS assay

*RAD54L* siRNA or negative control siRNA was transfected into KMS-28BM. Cells were seeded onto 96-well plates at a density of 2.0 × 10^4^ cells/well in 100 μl of culture medium. Cells were cultured for 24 h, 48 h and 72 h. At 0 h and after 24 h, 48 h and 72 h post-transfection, 20 μl of CellTiter 96 AQueous One Solution Reagent (Promega, USA) was added into each sample well. The plate was then incubated at 37 ℃ for 3–4 h in a humidified 5% CO_2_ incubator. Subsequently, the plate was measured at absorbances of 490 nm and 630 nm using Synergy HTX microplate reader (BioTek^®^, USA). The 630 nm reading was then subtracted from the 490 nm reading.

### Apoptosis assay

After 48 h of transfection, *RAD54L* siRNA and control siRNA-treated cells were harvested by centrifugation at 1000 rpm. Cells were washed with ice-cold PBS and then resuspended in Annexin binding buffer at 5.0 × 10^5^ cells/mL. This was followed by staining the cells with 5 μl of Annexin-V-FITC and 5 μl of propidium iodide (PI) (Elabscience, USA). Finally, the cells were analysed with the FACSCANTO II flow cytometer (BD BioScience, USA) in which 10,000 events were recorded for each analysis.

### Cell-cycle analysis

Briefly, transfection was performed according to the procedure described above. Cells transfected with *RAD54L* siRNA or control siRNA were then transferred to a 24-well cell culture plate containing pre-warm medium. The plate was then incubated at 37 °C CO_2_ cell incubator for 48 h. After 48 h of transfection, cells were harvested and washed with ice-cold PBS. This was followed by fixing the cells with 70% ethanol overnight. Approximately 1 × 10^5^ cells were stained with 500 μl of PI solution (BD BioScience, USA). The mixture was incubated for 15 min at room temperature. Cell cycle distribution was then analysed with FACSCANTO II flow cytometer (BD BioScience, USA) and ModFit LT (Verity Software House).

### The determination of RAD54L protein level with ELISA

Standards were diluted and prepared according to manufacturer’s protocol (SUNLONG Human RAD54L ELISA kit). Forty microliters of sample dilution buffer and 10 μl of sample were added into sample wells and then incubated for 30 min at 37 °C. Then, the wash buffer stock solution was diluted 1:30 in distilled water. Microtiter plate was washed for 30 s, repeatedly for 5 times. This was followed by adding 50 μl of HRP-conjugate reagent to each sample well. The mixture was then incubated and washed as described above. Then, 50 μl of each Chromogen Solution A and B were added into the sample well. Subsequently, the plate was incubated for 15 min at 37 °C. This was followed by adding 50 μl of stop solution to terminate the reaction. Lastly, absorbance was measured at 450 nm with a microplate reader (Tecan Infinite^®^ M1000).

### Statistical analysis

Student’s *t*-test was used to assess statistical significance between the means of the 2 groups. Those having *P*-value lower than 0.05 were considered significant.

## Results

### mRNA expression profiling and RT-qPCR verification

**Fig. 1:**
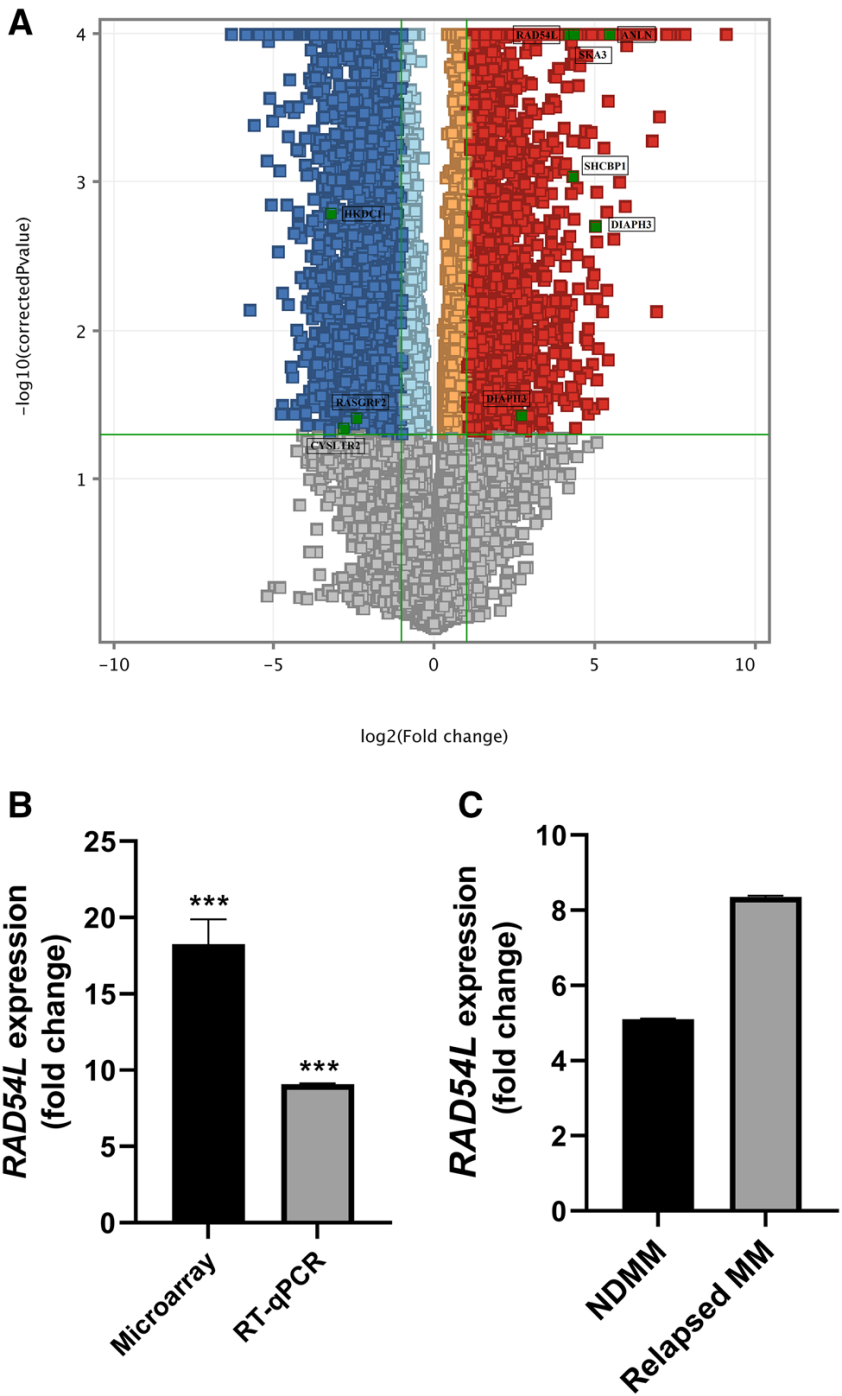
**A** Volcano plot identified 5888 differentially expressed probes in multiple myeloma vs. normal controls (fold change ≥ 2.0; *P* < 0.05). The *x*-axis represents the log_2_-fold change of probes, while the *y*-axis represents the − log_10_ of the corrected *P* values for each probe. Each dot represents a probe and the red- and blue-coloured areas represent the up-regulated and down-regulated probes, respectively, that met the selection criteria of a fold change of at least 2 (fold change ≥ 2.0 or ≤ 2.0) and a *P* < 0.05. Orange and light blue dots represent up-regulated and down-regulated probes that failed to pass the fold change cut-off, respectively. Green dots represent probes for *RAD54L*, *DIAPH3*, *SHCBP1*, *SKA3*, *ANLN*, *HKDC1*, *RASGRF2* and *CYSLTR2*. **B** Graph showing the mean expression of *RAD54L* detected in microarray and RT-qPCR. *RAD54L* is up-regulated in 23 MM samples as measured by RT-qPCR, consistent with microarray findings. **C** Graph showing mean expression of *RAD54L* in relapsed MM (N = 14) is higher than NDMM (N = 2) as determined by RT-qPCR. ***P < 0.001

mRNA expression profiling was performed for 19 MM samples, 7 MM cell lines and 3 normal controls. A total of 50739 probes were retained after normalising and filtering at selected thresholds. Out of 50739 probes, 5888 probes were significantly differentially expressed (*P* < 0.05; fold change ≥ 2.0). The results are depicted in a volcano plot (Fig. 1A). These 5888 probes were consisted of 5124 genes (including long intergenic non-protein coding RNAs, lincRNAs and novel transcripts). Among 5124 genes, 2696 and 2428 genes were up-regulated and down-regulated in MM vs. the control group, respectively. Genes showing up-regulation and down-regulation in MM vs. controls are listed in Online Resource 1 and Online Resource 2, respectively. Unsupervised hierarchical clustering was performed on up-regulated and down-regulated genes for all samples, respectively. Genes which expressed at similar patterns were clustered together and joined by a sequence of branches or dendrogram. The genes input list contained DEGs with a corrected *P* < 0.05 and a fold change of ≥ 2.0. Online Resources 3 & 4 showing the heatmaps of three distinct sub-clusters classified according to the degree of similarity in gene expression: MM cell lines, MM clinical samples (except MM9 for Online Resource 3; MM5 and MM9 for Online Resource 4) and controls.

The up-regulation of *RAD54L* as detected with microarray analysis was further verified by using RT-qPCR in 23 MM samples from the same cohort (except MM2, MM5 and MM9, where the RNA concentration was insufficient for RT-qPCR). The RT-qPCR results showed that *RAD54L* was significantly up-regulated in the MM vs. control group, consistent with microarray findings (*P* < 0.001) (Fig. 1B). Additionally, when we compared the relative expression of *RAD54L* in NDMM (N = 14) with the relapsed MM (N = 2), the RT-qPCR findings showed that *RAD54L* expression in relapsed MM was higher than NDMM, although the result was not significant (Fig. 1C).

### The effects of *RAD54L* gene silencing on cell proliferation, apoptosis, cell cycle distribution and protein level in KMS-28BM MM cells

**Fig. 2:**
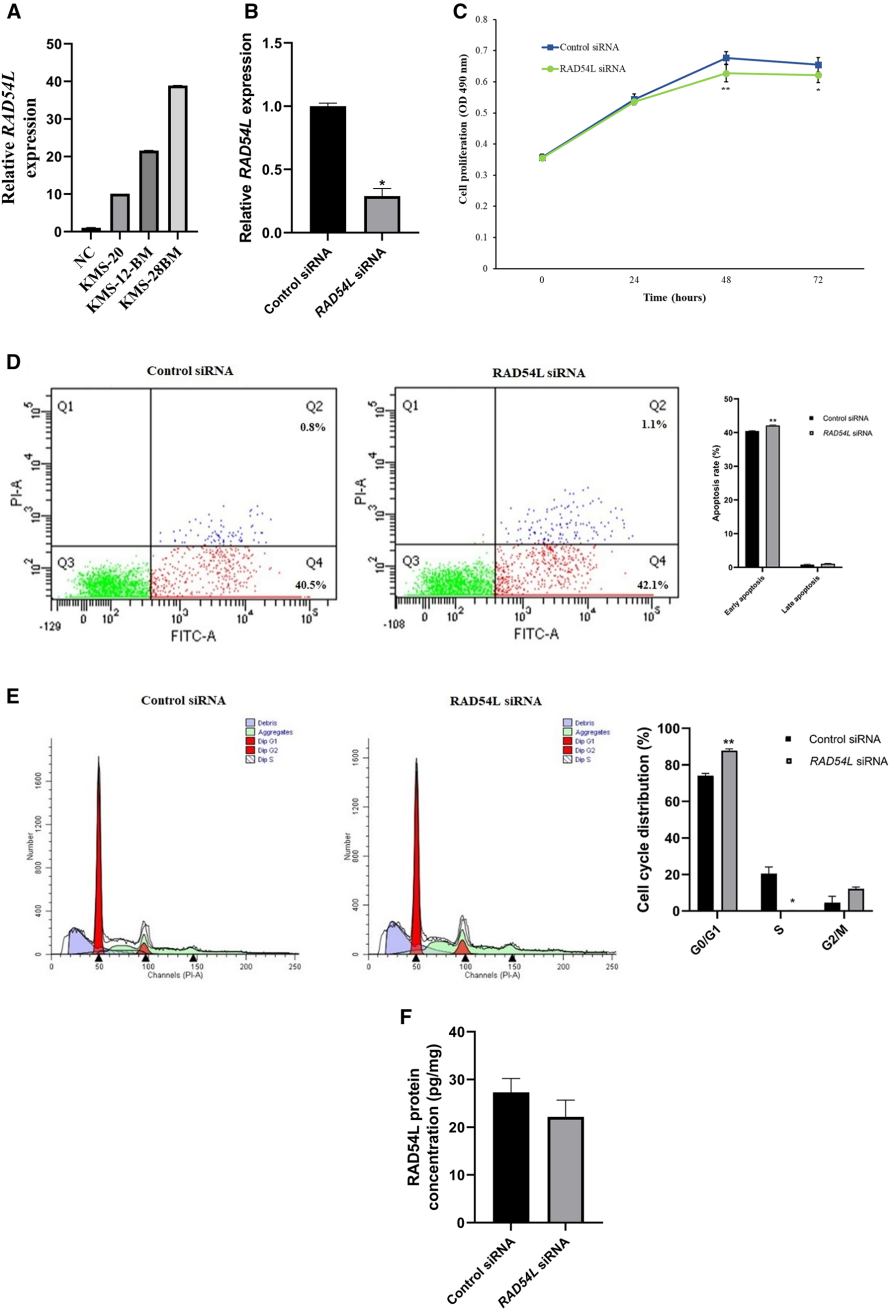
**A** Graph showing relative *RAD54L* expression in KMS-20, KMS-12BM and KMS-28BM MM cell lines as measured by RT-qPCR. **B** Relative *RAD54L* expression was reduced by 71% in *RAD54L* siRNA-treated vs. control siRNA-treated KMS-28BM cells at 24 h post-transfection as measured by RT-qPCR. **C** Cell proliferation was decreased in KMS-28BM cells transfected with *RAD54L* siRNA compared to control siRNA at 48 h and 72 h post-transfection as analysed by MTS assay. **D** Number of early apoptotic cells showing small but significant increase in KMS-28BM cells transfected with *RAD54L* siRNA compared to control siRNA at 48 h post-transfection as analysed by flow cytometry. **E** *RAD54L* gene silencing significantly induced cell cycle arrest at G0/G1 phase while reduced S phase in *RAD54L* siRNA-treated cells compared to control at 48 h post-transfection as determined by flow cytometry. **F** RAD54L protein expression level was decreased by approximately 19% in KMS-28BM cells transfected with *RAD54L* siRNA compared to control siRNA at 48 h post-transfection as measured by ELISA assay. *P < 0.05; **P < 0.01

We have previously integrated the gene expression profiles with the miRNA expression profiles from the matched MM samples and revealed an inverse correlation between 5 putative target genes (*RAD54L*, *CCNA2*, *CYSLTR2*, *HKDC1* and *RASGRF2*) and 15 dysregulated miRNAs (Bong et al. 2017). Among the 5 genes listed, *RAD54L* is one of the new genes that has never been reported in association with MM. This prompted us to explore the in vitro function of *RAD54L* in MM cells. Since the MM clinical samples used in this study were derived from the Asian population, we would like to study the function of *RAD54L* in MM cell lines derived from the same origin. Thus, we have selected KMS-28BM as a model for functional analysis: a cell line which showed the highest *RAD54L* expression level among the 3 cell lines derived from Asian patients included for the gene expression profiling (Fig. 2A).

Silencing of *RAD54L* gene with siRNA duplexes successfully knockdown *RAD54L* expression in KMS-28BM by 71% at 24 h post-transfection as measured by RT-qPCR (*P* < 0.05) (Fig. 2B). The MTS assay revealed significant decreased in proliferation of MM cells treated with *RAD54L* siRNA compared to the control siRNA at 48 h (*P* < 0.01) and 72 h (*P* < 0.05) post-transfection (Fig. 2C). Flow cytometry results showed that silencing of *RAD54L* led to a small but significant increase in the numbers of early apoptotic cells in siRNA-treated cells compared to the control siRNA-treated cells (*P* < 0.01) (Fig. 2D). Additionally, cell cycle analysis showed that gene silencing of *RAD54L* significantly induced cell cycle arrest at G0/G1 phase (*P* < 0.01) while reduced S phase (*P* < 0.05) in KMS-28BM (Fig. 2E). ELISA results showed that RAD54L protein expression diminished by approximately 19% with *RAD54L* knockdown at 48 h post-transfection (*P* < 0.05) (Fig. 2F).

## Discussion

MM is a highly heterogeneous and complex disease which develops via a stepwise process involving multiple genetic aberrations. Numerous genetic aberrations have been identified in association with MM development and malignancy. However, to date MM is still an incurable disease mainly because most of the patients eventually relapse or are refractory to the available treatments (Davis and Sherbenou 2021). Thus, identification of new molecular targets is urgently needed to solve this problem.

Herein, we used mRNA expression profiling to identify potential genes involved in the molecular pathogenesis of MM. Unsupervised hierarchical clustering analysis of the up-regulated and down-regulated probes clearly clustered the samples into MM cell lines, MM clinical samples (except MM9 for the up-regulated probes and MM5 & MM9 for the down-regulated probes) and control groups (Online Resources 3 & 4). This scenario depicts that gene expression changes occur during the transition from normal to malignant plasma cells (Szalat et al. 2016). This study identified a total of 5124 DEGs in the MM vs. control group. Our findings revealed up-regulation of recurrent genes involved in primary oncogenic events: *CCND1* and *FGFR3* (Kuehl and Bergsagel 2012). Prominent and potential genes involved in the secondary oncogenic event such as *NRAS*, *IRF4*, *IDH2*, *PSMB5* and *APOBEC2* were found to be up-regulated whereas *SP140*, *LTB* and *ATM* were down-regulated in MM (Kuehl and Bergsagel 2012; Bolli et al. 2014; Walker et al. 2018; Allmeroth et al. 2021). Apart from that, differential expression of genes involved in NF-κB pathway were identified (*NFKBIB*, *IKBKB*, *CARD11*, *TNFRSF1A*, *MAP3K1*, *MAP3K14* and *TLR4*) suggesting the important role of NF-κB pathway in myelomagenesis (Chapman et al. 2011). Aberrant expression of apoptosis-related genes such as *BCL2*, *BIK* and *BAX* were also detected in the current study (Gupta et al. 2021). Additionally, the most established growth factors and cytokines involved in bone marrow microenvironment, namely *IL6* and *IGF1*, were found to be over-expressed in the MM vs. control group in this study (Birmann et al. 2009). Other DEGs, namely *BIRC5*, *CENPA*, *CCNB1*, *CHEK1*, *AURKB*, *BUB1*, *BUB1B*, *NEK2*, *ASPM*, *TOP2A* and *EZH2* have been described elsewhere and they play a role, at least in part, in the molecular pathogenesis of MM (Chng and Fonseca 2009; Broyl et al. 2010; Chung et al. 2013). Our findings revealed that most of the DEGs are involved in DNA repair, cell proliferation, cell cycle and mitotic/spindle checkpoints, mismatch repair pathway, kinetochore and microtubule attachment and NF-κB pathway (Chng et al. 2006; Ueki et al. 2008; Chng and Fonseca 2009; Ruiz et al. 2009; Broyl et al. 2010; Agarwal et al. 2011; Chapman et al. 2011; Asano et al. 2013; Bengtsson et al. 2013; Chung et al. 2013; Jiao et al. 2013; Li and Huang 2013; Chuang and Ou 2014; Morley et al. 2015; Perez-Peña et al. 2017; Contreras et al. 2019; Ji et al. 2019; Xia et al. 2020; Borah and Reddy 2021; Li et al. 2021). DEGs and their functions are shown in Table 2.

**Table 2:** Functions and fold changes of differentially expressed genes (DEGs)

Function	Gene symbol (fold change)
Cell cycle and cell cycle checkpoint	*CCNA2* (20.6), *BIRC5* (32.0), *CENPA* (39.0), *CENPF* (18.4), *CCNB1* (10.5), *CCNB2* (33.0), *CDC25C* (19.0), *KIF14* (29.1), *DEPDC1* (13.3), *CDK1* (18.7), *CCND1* (7.8)
DNA repair	*RAD54L* (18.3), *RAD51AP1* (26.0), *RAD51* (13.8), *CHEK1* (11.2), *ATM* (-3.5)
Mitotic/spindle checkpoints	*PLK1* (17.1), *NUF2* (17.7), *AURKB* (20.9), *CDC20* (11.8), *BUB1* (18.7), *BUB1B* (24.0), *CENPA* (39.0), *NEK2* (21.8), *TTK* (41.1), *TK1* (23.2), *CKAP2L* (43.0), *KIF11* (14.7), *KIF20A* (35.0), *SKA1* (40.8), *TPX2* (22.3), *DTL* (33.1)
Cell proliferation	*ASPM* (27.2), *TOP2A* (23.8), *TTK* (41.1), *E2F1* (14.2), *E2F7* (18.5), *E2F8* (33.4), *CDCA8* (13.4), *SHCBP1* (19.6), *FGFR3* (20.2), *PSMB5* (2.2), *ATM* (-3.6)
Kinetochore and microtubule attachment	*ZWINT* (13.6), *AURKB* (20.9), *BIRC5* (32.0), *CENPA* (39.0), *TTK* (41.1), *KIF2C* (27.0), *SKA3* (20.2)
Apoptosis	*BCL2* (− 2.9), *BIK* (3.6), *BAX* (2.2)
Growth factor	*IL6* (15.6), *IGF1* (9.2), *IGF2* (18.6)
Mismatch repair pathway	*PCNA* (5.9)
Centrosome	*KIF11* (14.7), *KIF15* (20.2), *AURKB* (20.9)
Chromatin regulator	*SP140* (− 3.1)
Ubiquitin proteasome	*UBE2S* (6.1), *UBE2T* (11.5)
Cytokine	*IRF4* (3.3)
Metabolism	*HKDC1* (− 9.3), *IDH2* (2.2)
Cytoskeleton	*DIAPH3* (19.6), *ANLN* (44.1)
TNF-associated gene	*LTB* (− 9.6)
Polycomb	*EZH2* (5.6)
NFκB pathway	*IKBKB* (− 3.1), *NFKBIB* (2.0), *CARD11* (− 2.9), *TNFRSF1A* (− 4.5), *MAP3K1* (− 2.0), *MAP3K14* (− 2.3), *TLR4* (− 6.8)
RAS related pathway	*NRAS* (2.3), *RASGRF2* (− 5.5)
CysLT signaling	*CYSLTR2* (− 7.3)
APOBEC-associated genes	*APOBEC2* (2.6)*, APOBEC3G* (− 2.1)
Histone	*HIST1H2BI* (2.3)*, HIST1H2BK* (2.1)*, HIST1H2BL* (2.1)*, HIST1H2BM* (2.1)*, HIST1H2BB* (2.8)*, HIST1H2BD* (2.1)*, HIST1H2BE* (3.1)*, HIST1H2BF* (5.4)*, HIST1H2BG* (2.5)*, HIST1H2BH* (2.1)*, HIST3H2BB* (2.8)*, HIST1H3H* (3.5)*, HIST2H3A* (42.6)*, HIST1H3B* (9.6)*, HIST1H3D* (4.0)*, HIST1H3F (*4.3)*, HIST1H3G* (2.4)*, HIST1H4A* (3.6)*, HIST1H4K* (2.3)*, HIST1H4L* (3.7)*, HIST1H4B* (3.5)*, HIST1H4C* (3.1)*, HIST1H4D* (3.9)

Interestingly, our study reveals significant DEGs, which have never been reported in association with myelomagenesis. They are *RAD54L*, *DIAPH3*, *SHCBP1*, *SKA3*, *ANLN, HKDC1*, *RASGRF2* and *CYSLTR2*. The *RAD54L*, *DIAPH3*, *SHCBP1*, *SKA3* and *ANLN* were up-regulated while *HKDC1*, *RASGRF2* and *CYSLTR2* were down-regulated in MM vs. controls. Over-expression of *RAD54L* was detected in the MM vs. control group in this study by 18.3-folds. We then verified the expression of *RAD54L* in 23 samples by using RT-qPCR and the results are consistent with microarray analysis. *RAD54L* is involved in homologous recombination repairing of DNA double-strand breaks to facilitate human genomic integrity and genetic diversity (Andriuskevicius et al. 2018). Defects in homologous recombination pathway-related genes *RAD54*, *RAD51* and *RAD52* could lead to tumour development (Mun et al. 2020). RAD54 interacts with RAD51 nucleoprotein filament to form RAD54-RAD51-ssDNA nucleoprotein complex to stimulate homology search and DNA strand exchange (Zohud et al. 2020). RAD54 also removes RAD51 from heterodimeric DNA in an ATP-dependent manner after DNA strand exchange (Andriuskevicius et al. 2018; Rosenbaum et al. 2019). In G2 phase of the cell cycle, RAD54 is phosphorylated to remove RAD51 to facilitate homologous recombination while it is not phosphorylated in S phase to allow RAD51 stabilizing and protecting the stalled replication forks from nucleolytic degradation (Spies et al. 2016). Thus, *RAD54* deficiency reduces homologous recombination efficiency. In addition to *RAD54L*, our results revealed up-regulation of *RAD51* (13.8-folds), implicating that the deficiency in the homologous recombination pathway plays a critical role, at least in part, in the pathogenesis of MM.

Elevated expression of *RAD54L* is detected in carcinomas of the breast, colon, lymphoma and meningioma; however, its role in MM pathogenesis is unknown (Leone et al. 2003). In colorectal carcinoma, *RAD54L* taking part in maintaining chromosomal stability via DNA homologous recombination and p53 signalling pathway by interacting with POLE (Zohud et al. 2020). Apart from p53 pathway, *RAD54L* also interacting with other genes such as *E2F1* and *NEK2* in tumourigenesis (Mun et al. 2020; Pavan et al. 2021). Interestingly, *E2F1* and *NEK2* were found to be up-regulated in the MM vs. control group in this study by 14.2 and 21.8-folds, respectively. *E2F1* is a well-recognized transcription factor that regulates cell cycle progression in the suppression of tumourigenesis, whereas *NEK2* is involved in maintaining the stability of replication forks (Karras et al. 2016; Pavan et al. 2021).

The p53-DREAM pathway is a newly described p53-mediated cell cycle arrest pathway. The p53-DREAM pathway regulates not only cell cycle associated genes essential for cell cycle progression from G1 phase to the end of mitosis, but also DNA repair and telomere maintenance genes (Fischer et al. 2014; Engeland 2018). Thus, defects in this pathway lead to the loss of checkpoint control and uncontrolled cell division (Engeland 2018). Apart from that, aberrant expression of genes in this pathway induces chromosomal instability and aneuploidy in cancer cells (Thompson and Compton 2010). To date, almost 250 target genes regulated by the p53-DREAM pathway have been identified (Fischer et al. 2014; Engeland 2018). Interestingly, *RAD54L* and many other DEGs that function in cell cycle or checkpoint control identified in the current study are components of the p53-DREAM pathway (*CCNA2*, *BIRC5*, *CENPA*, *CENPF*, *CCNB1*, *CCNB2*, *CDC25C*, *KIF14*, *DEPDC1* and *CDK1*) (Table 2) (Fischer et al. 2014; Engeland 2018). *CCNA2*, *BIRC5*, *CCNB1*, *CCNB2*, *CDC25C* and *CDK1* are key regulators in the p53-DREAM pathway (Fischer et al. 2015, 2016). It was postulated that *RAD54L*, *CCNA2* and *CCNB1* are p53-dependent repression of the cell cycle genes, in which they contain cell cycle genes homology region (CHR) elements that allow them to bind with DREAM transcriptional repressor (Fischer et al. 2014, 2016). Most of these target genes are involved in G2/M checkpoint control and progression through mitosis (Fischer et al. 2016). Our results indicate that disruption of the p53-DREAM pathway plays a critical role in MM pathogenesis as many genes involved in this pathway were differentially expressed in MM.

In terms of epigenetic aspect, our previous findings on the integrative analysis of the mRNA and miRNA expression profiling for matched samples revealed an inverse correlation between *RAD54L* and cancer-associated miR-150 (Bong et al. 2017). Based on computational predictions, our previous findings revealed that differential expression of *RAD54L* might be correlated with aberrant expression of miR-150-5p, which regulates the gene. Thus, according to our observation, we suggest that *RAD54L* possibly triggers MM either independently or by interacting with other genes/miRNA via impairing DNA repair mechanism and cell cycle progression.

To validate the function of *RAD54L* in MM, we transiently knockdown *RAD54L* in KMS-28BM human MM cell line using siRNA method. The effects of *RAD54L*-mediated gene silencing on cell proliferation, apoptosis and cell cycle distribution in KMS-28BM were determined. Our results reveal that gene silencing of *RAD54L* inhibits cell proliferation in KMS-28BM (Fig. 2C). In addition, *RAD54L* knockdown induces small but significant changes in early apoptotic cells (Fig. 2D). Moreover, cell cycle analysis demonstrates that depletion of *RAD54L* induces cell cycle arrest at G0/G1 phase and inhibits cells at S phase suggesting that *RAD54L* is involved in cell cycle progression (Fig. 2E). The ELISA assay shows that silencing of *RAD54L* not only knockdown the gene expression at the mRNA level but also decreases its protein expression level (Fig. 2F). For the first time, our results demonstrate that up-regulation of *RAD54L* might play a key role, at least in part, in MM pathogenesis by activating myeloma cell growth, inhibiting apoptosis and impairing cell cycle progression.

The limitation of the present study is the small number of samples used for gene expression profiling, which may reduce the statistical power of the study. Our future direction is to validate the results with larger sample sizes by using a more advanced technology such as RNA-sequencing (RNA-seq).

## Conclusions

This study has identified possible molecular targets underlying MM pathogenesis. *RAD54L* might be a potential therapeutic target in MM, possibly functioning in the cell cycle and checkpoint control.

## Supplementary Information

Below is the link to the electronic supplementary material.

Supplementary file1 Significant up-regulated probes identified by mRNA expression profiling (≥2.0 fold change, *P* < 0.05) (XLSX 243 KB)

Supplementary file2 Significant down-regulated probes identified by mRNA expression profiling (≥2.0 fold change, *P* < 0.05) (XLSX 225 KB)

Supplementary file3 Heatmap showing up-regulated probes and detailed view of the newly identified probes RAD54L, DIAPH3, SHCBP1, SKA3, ANLN (fold change ≥2.0; *P* < 0.05) (TIF 1037 KB)

Supplementary file4 Heatmap showing down-regulated probes and detailed view of the newly identified probes HKDC1, RASGRF2 and CYSLTR2 (fold change ≥2.0; *P* < 0.05) (TIF 1035 KB)

## Data Availability

The microarray-generated gene expression data can be retrieved from NCBI Gene Expression Omnibus (GEO) repository as series accession identifier GSE72213.
