# Supplementary figures and images for: Gene expression profiling and in vitro functional studies reveal RAD54L as a potential therapeutic target in multiple myeloma
Source: Genes Genomics. 2022 Jun 11;44(8):957–66. doi: 10.1007/s13258-022-01272-7 (PMC9273556; doi:10.1007/s13258-022-01272-7)

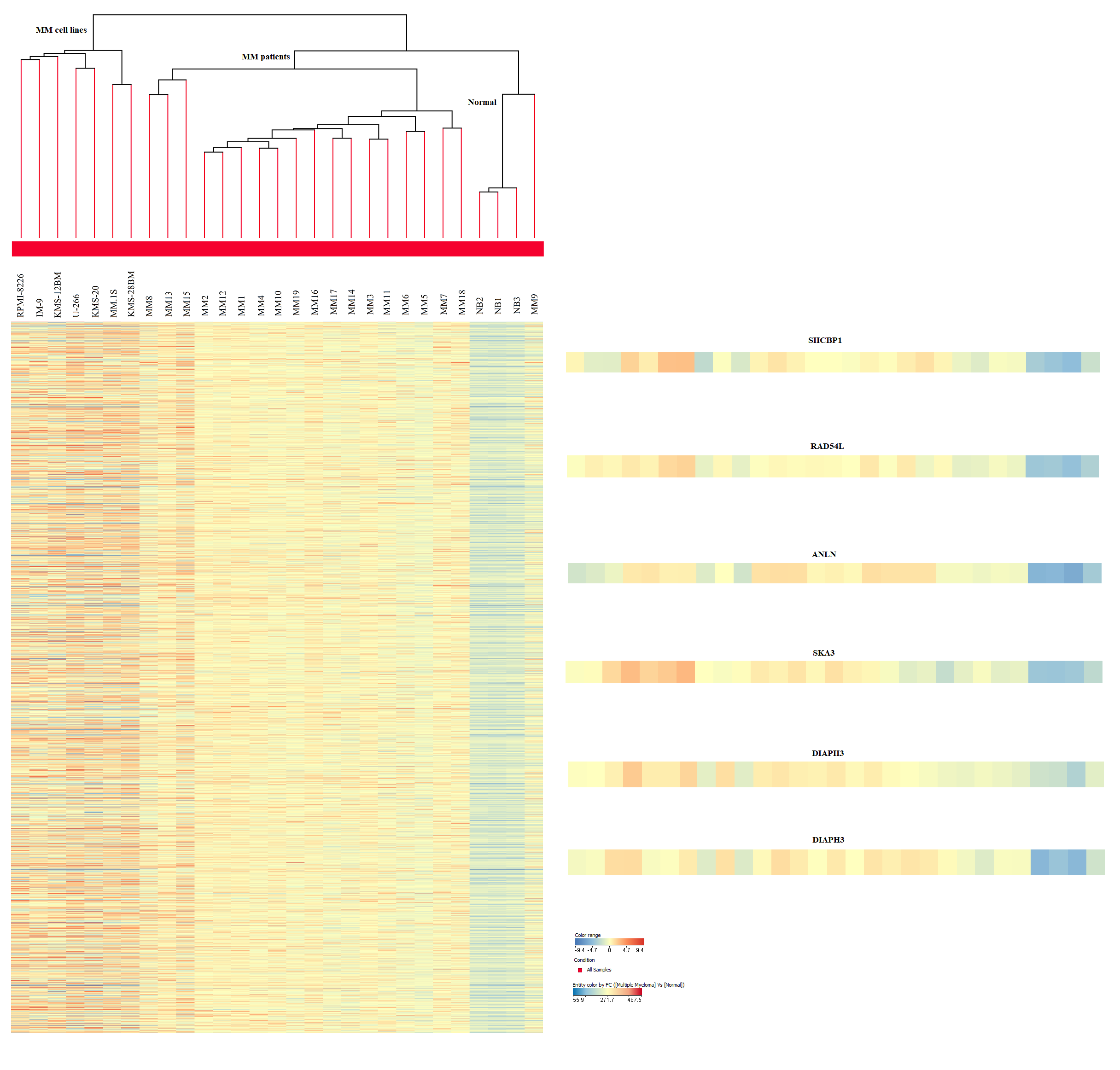

Supplement: Supplementary file 3 — Supplementary file3 Heatmap showing up-regulated probes and detailed view of the newly identified probes RAD54L, DIAPH3, SHCBP1, SKA3, ANLN (fold change ≥2.0; P < 0.05) (TIF 1037 KB) [file 13258_2022_1272_MOESM3_ESM.tif]

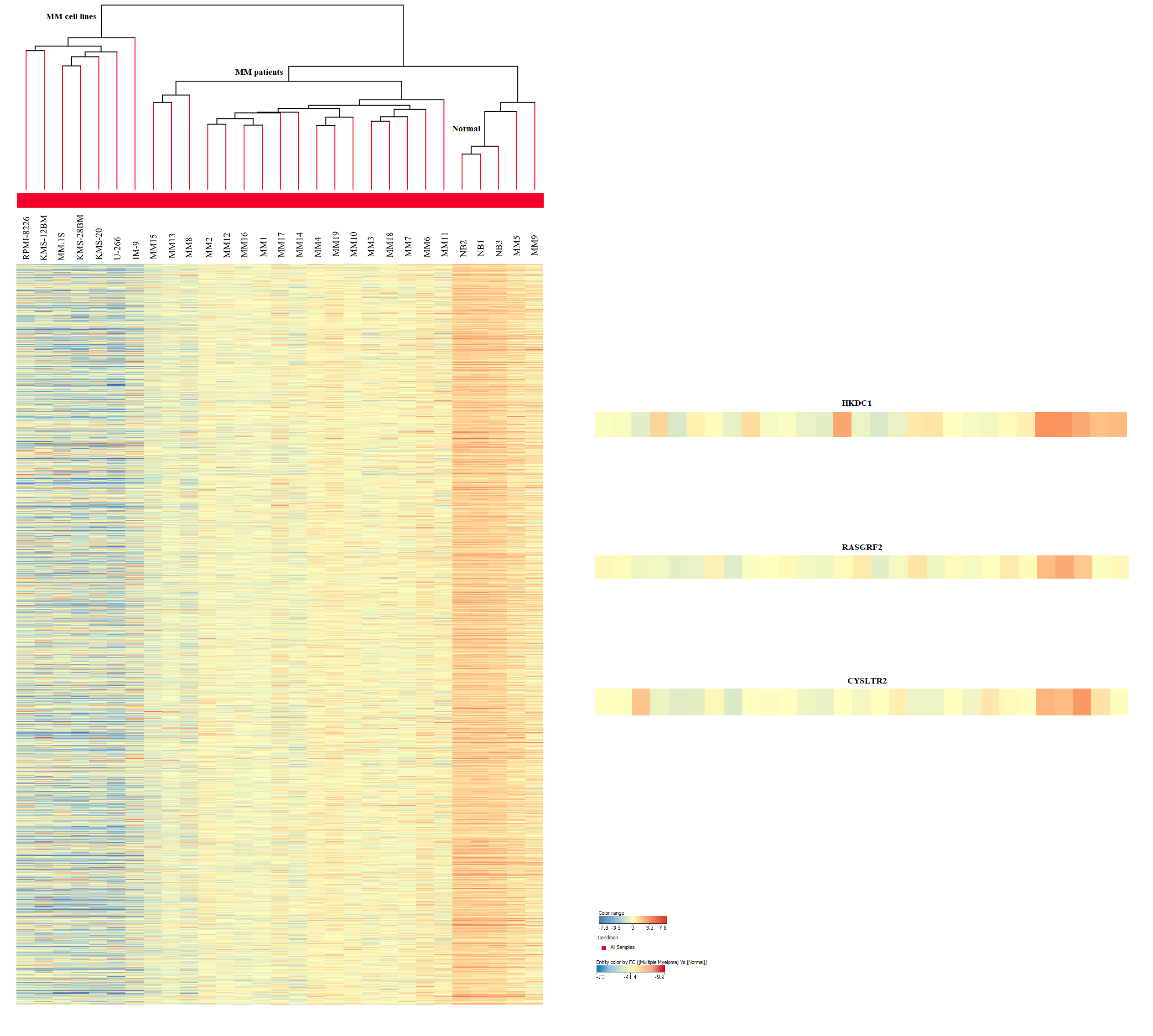

Supplement: Supplementary file 4 — Supplementary file4 Heatmap showing down-regulated probes and detailed view of the newly identified probes HKDC1, RASGRF2 and CYSLTR2 (fold change ≥2.0; P < 0.05) (TIF 1035 KB) [file 13258_2022_1272_MOESM4_ESM.tif]
